# “Their Packaging Has Always Been Like a Power”: A Qualitative Study of U.S. Smokers’ Perceptions of Cigarette Pack Visual Design Features to Inform Product Regulation

**DOI:** 10.3390/ijerph14101234

**Published:** 2017-10-17

**Authors:** Joseph G. L. Lee, Paige E. Averett, Tiffany Blanchflower, Nunzio Landi, Kyle R. Gregory

**Affiliations:** 1 Department of Health Education and Promotion, College of Health and Human Performance, East Carolina University, Greenville, NC 27858, USA; nunzioalandi@gmail.com (N.L.); kyle.ryan.gregory@gmail.com (K.R.G.); 2 School of Social Work, College of Health and Human Performance, East Carolina University, Greenville, NC 27858, USA; averettp@ecu.edu; 3 Department of Interior Design and Fashion Merchandising, College of Health and Human Performance, East Carolina University, Greenville, NC 27858, USA; blanchflowert15@ecu.edu

**Keywords:** marketing, product packaging, smoking, United States Food and Drug Administration, tobacco products

## Abstract

Cigarette packaging matters to consumer behavior. However, it is less clear which changes to packaging design would be salient for adult smokers. Such information is critically important to regulators in the United States who are charged with reviewing new tobacco products for their impact on population health. In this qualitative study, U.S. adult smokers (*n* = 33) participated in six telephone-based focus groups in March 2017. Separate groups were comprised of lesbian, gay, and bisexual (LGB) participants; participants with less than four years of post-secondary education; a mix of LGB and straight participants; and, the general population. All groups were purposely selected for diversity. Open thematic coding identified salient design elements used on cigarette packaging. Smokers articulated design elements’ use, meaning, and links with consumer behaviors. Three themes were identified: (1) the power of color, (2) supporting color with other design elements (e.g., logos/images, typography, the pack itself), and (3) the combined product brand experience of multiple design elements. Participants linked design elements to product characteristics and to consumer behavior (e.g., purchase). As the Food and Drug Administration is charged with regulating tobacco products, these findings suggest the importance of considering the cigarette pack part of the characteristics of a product.

## 1. Introduction

The visual design of cigarette packaging conveys brand identity and information about the product to consumers [1,2]. The impact of package design is well established in marketing, graphic design, and studies of food packaging [3,4,5]; it is also clear in tobacco industry documents [6,7,8,9]. Changes to design elements of cigarette packaging (e.g., color, type, logos) can alter consumption among different groups by targeting specific subpopulations (e.g., women [10] and adolescent girls [11]). Internal tobacco industry research shows cigarette packaging changes are designed to influence consumption patterns by making certain products more appealing to specific consumer groups in ways that can increase profits [6,7,12].

In the U.S., the Food and Drug Administration (FDA), with the 2009 signing of the Family Smoking Prevention and Tobacco Control Act (TCA), became responsible for regulation of tobacco products. This includes the review and approval of all new tobacco products based on available medical, scientific, and other technological evidence as appropriate for the protection of the public’s health. This public health standard is determined with respect to the risks and benefits to the population as a whole, including users and non-users of tobacco products [13]. The TCA created a new field of tobacco regulatory science, which the FDA charged with providing evidence for the development and implementation of regulations of tobacco products [14,15]. The FDA’s public health framework for tobacco regulation lists key elements of interest in tobacco regulatory science, and this study responds to Element 2 of that framework: “Restrict product changes to protect public health” (p. 1047) [14].

In the product design literature, a new product is often the extension of an existing product and draws on brand identity while also differentiating itself in some way [12,13]. New products are defined by the FDA as those not marketed as of 15 February 2007, or as modified versions of products available prior to that date that are not substantially equivalent to the predicate product. Thus, new (or altered) products entering the market must now go through one of two pathways: one, pre-market review to ensure the product meets the public health standard (e.g., its influence on users and nonusers to assess net population-level health impacts) or, two, demonstrate substantial equivalence to a predicate product.

Guidance released by the FDA in 2015 indicated that changes in the visual design of an existing product’s packaging would be subject to review, as such changes constituted a new tobacco product [16]. Litigation challenging this interpretation ensued with the U.S. District Court for the District of Columbia ruling that Congress did not intend for changes to the visual design of existing packages to constitute a “new tobacco product” [16]. However, the FDA has since stated that changes to tobacco packaging should be considered part of the tobacco product when it is reasonably expected to alter or affect the tobacco product’s performance or characteristics [17]. Thus, it remains critically important that the FDA have a strong scientific basis for identifying which cigarette packaging design elements are most salient and influential in shaping consumer perceptions. Changes to these elements on cigarette packs could influence population health.

The link between the visual design of consumer products and consumer behavior is well theorized and supported [5,18]. Our work was guided by the Context of Consumption Framework, which posits that the sensory experience of a product mediates the relationship between the design of the product and consumer response [5]. Responses are conceptualized into three cognitive and five affective domains. The cognitive responses include (1) responses to the novelty or aesthetics of the product; (2) what the design signifies about the qualities of the product within; and (3) what the design says about the social identity of the user. Affective responses include (1) emotional responses to the function of the product; (2) its design; (3) its desirability; (4) its social connotations; and (5) its ability to garner interest [5]. It is important to note that the underpinning of this design-based framework is semiotics, which is also known as the study of meanings given to signs and symbols (e.g., the design features of a product) [18].

More specifically, research has documented that visual design of products has influence on consumers’ perceptions and consumption behaviors [18,19,20]. Color is particularly influential [6,21,22,23,24]. However, graphic design research provides evidence that packaging encompasses a larger range of design elements [19] that may be amenable to FDA regulation. As such, we sought to qualitatively identify what smokers view as salient cigarette packaging design elements. Findings from this study critically inform FDA efforts to identity design elements appropriate for regulations to protect population health.

## 2. Materials and Methods 

### 2.1. Methods and Instrument

The research protocol was approved by the East Carolina University and Medical Center IRB (#16-001200). Following the best practices in tobacco regulatory science [15], our research team included a lawyer specializing in tobacco regulatory science (Kyle R. Gregory). Adult smokers were recruited from the National Opinion Research Center (NORC) at the University of Chicago’s AmeriSpeak Panel to participate in six telephone-based focus groups. Telephone groups were utilized in order to bring together people across a wide geographical area [25]. AmeriSpeak is a probability-based and nationally-representative panel. AmeriSpeak staff recruited participants to maximize diversity in two groups comprised of lesbian, gay, and bisexual (LGB) participants; two groups comprised of participants with fewer than four years of post-secondary education; one group comprised of a mix of LGB and heterosexual smokers; and, one group of the general population of smokers. We emphasized less formal education and LGB identities given heightened smoking prevalence among both populations [26]. Focus groups, which took place in March 2017, were moderated by an experienced NORC staff member after participants provided consent. Audio recordings were professionally transcribed using a smooth verbatim protocol. Transcripts were not returned to participants for comments. Two authors attended each group (Joseph G. L. Lee, Paige E. Averett) and confirmed a saturation of themes. Participants were compensated in AmeriSpeak points. We follow a qualitative study reporting checklist [27].

We piloted, and then used, a semi-structured focus group guide designed to prompt discussion of cognitive, affective, and behavioral connections with cigarette packaging design elements. The guide (Online Supplemental File 1) was informed by a theory-based model of the role of visual product design on the consumer behaviors described above [5].

### 2.2. Participants

The six focus groups included 33 participants; four of whom left the group before completion. Reasons reported for leaving early were not related to the study content, e.g., having an appointment. Participants lived in all nine U.S. Census divisions, indicating wide geographic representation. Just under half of the 33 identified as White (49%) with fewer identifying as Black (24%), Hispanic (9%), American Indian (3%), or multi-racial (15%). Most were female (64%), and participants had an average age of 45 (SD = 11.5, range 22–62). All participants were classified as current smokers (6% reporting smoking “some days” and 94% reporting “every day”), 46% usually smoked menthol (nationally, over a third of smokers usually smoke menthol [28]), and 58% smoked their first cigarette within 30 minutes of waking. Just under 20% of participants had no Internet access at home (18%), and 49% had fewer than four years of postsecondary education.

### 2.3. Analysis

Notes from the focus group were compiled and discussed after the groups by two authors (Paige E. Averett, Joseph G. L. Lee). Focus group data were analyzed via the constant comparison analysis method as developed by Glasser and Strauss [29]. This method involves three stages: open coding, axial coding, and selective coding. In open coding, data were first sorted into small units by the lead analyst (Paige E. Averett, who holds a PhD in human development, is a professor of social work, and specializes in qualitative methodology), and, with feedback from the team members, a descriptor or code was attached to each of the units. The other analysts brought additional perspectives, including a PhD in public health and expertise in tobacco control (Joseph G. L. Lee), a PhD in consumer behavior and expertise in brand identity (Tiffany Blanchflower), and a BFA in graphic design (Nunzio Landi). Two team members had experience smoking cigarettes, but none were current smokers. During axial coding, the initial codes were grouped together in meaningful ways, looking for similarity and overlap. Four authors conducted repeated review of the coding during the axial coding stage. In the final selective coding stage, we further grouped the data and interpreted larger meanings. In both axial and selective coding, disagreements in coding between the four authors were resolved by discussion and consensus. Due to the use of NORC’s AmeriSpeak Panel, we were unable to complete member checks. In the final analysis, the research team focused on the themes that best represented the data: the power of color, supporting color with other design elements, and product brand experience.

## 3. Results

### 3.1. The Power of Color

Cigarette packaging colors emerged as a very strong and consistent theme for our participants, and appear to be the most salient design element for these groups of smokers. Participants discussed color even when talking about other design elements and often as the primary descriptor of their cigarette packs.

“The first pack was gold and white. They were Marlboro Lights. I’ve moved on to Reds since then.”

(Group 5, Lower Education)

Package color was also pointed out as a very important aspect of cigarette packages regardless of other design elements:

“When I think about the initial packaging, that was the red and white package.”

(Group 1, LGB)

“The color. The yellow. The whole pack was yellow.”

(Group 4, Mix)

Additionally, the analysis identified several subthemes including emergence of color in discussions of other topics, the role of color in relation to taste/strength, and the importance of unique colors.

### 3.2. Color Even When Not Discussing Color

Although participants often outright discussed the importance of color, the data showed that color was also a latent design feature. For instance, when asked about brands they currently smoke or the brand that they began smoking, participants would state the brand but immediately connect the brand and packaging with a particular color.

“She smoked Winston 100s in the gold pack.”

(Group 1, LGB)

“Well, I myself smoke Marlboros Red in the box and I started smoking them when I was real young.”

(Group 2, Lower Education)

### 3.3. Color Represents Taste and Strength

Participants explained that color influenced the way that they purchase cigarettes, for example:

“Because that’s how you have to describe them when you buy them from a retailer is by the color is what the brand is which of course there’s turquoise and green is menthol. Blue is the light and then dark blue, there’s like a light blue and then dark blue is like the regular type cigarette like a Marlboro and then the yellow is another version of the light but it’s like the organic kind.”

(Group 2, Lower Education)

In this typical example, package color signaled to consumers the strength of the products. Additionally, colors on packages were often how smokers discern the differences in taste or flavor:

“I think the biggest change is the brands took away like the full strength and the lights and ultralights and menthol. They did away with those words and now it’s just indicated by color which I know at first it was a little weird.”

(Group 5, Lower Education)

### 3.4. Being Unique with Color

Given the importance of color, it is not surprising that different or unique colors used in packaging influenced participants’ perceptions of various cigarette brands. Participants often discussed cigarette packaging colors that were novel or unique as ones they were drawn to:

“All the other colors like white and red and green—those boxes kind of all look alike. They look the same. The black and gold, my eye just directly goes straight to it.”

(Group 3, LGB)

“I would say a lot is when the brand changes something. Like if you smoked Marlboros and when they first started, came out with the Marlboro black, you’re used to their normal line up of color packages. You see them, and then all of a sudden there’s this black pack in the midst of them. Oh, well, what, what’s that? You know?”

(Group 4, Mix)

Color was the most important aspect of package design to our participants and plays a central role in how participants describe the product packaging. Moreover, color was a design feature participants relied on to identify differences in taste and flavor. Color is a primary and salient design feature used in cigarette packaging design.

### 3.5. Supporting Color with Other Design Elements

In addition to color, participants discussed other package design elements, including logo and image, typography, and the pack itself. Often these design elements were linked with color and played a supporting role in participants’ interpretation of the package design.

#### 3.5.1. The Power of Graphics: Logos and Images

In addition to color, participants also described the logos and images on the cigarette packages as important design elements. Participants noted that the symbols on the packages were an eye-catching design element they were drawn to.

“I always liked the Newport packaging because of the upside Nike symbol.”

(Group 2, Lower Education)

“There’s like an Indian eagle and a round circle, red circle that’s kind of like a sun with an Indian silhouette in it, smoking a pipe.”

(Group 6, General Population)

Beyond finding them eye-catching, participants shared that they associated some logos and images with meanings that extended beyond the brand identity of some cigarette brands.

“And I know that Newports, that’s one of the things they pushed is that they have a lower tar content than all the other cigarettes do so that’s kind of a checkmark that then this is a good thing.”

(Group 3, LGB)

“I guess when you think of the camel, you think of like the desert, so you think of a calm place. Maybe that’s not such a calm place. Dry. But you think of—I think of the sun and it’s like it’s just more calm. I would think that it would be more calm than harsh on your lungs.”

(Group 3, LGB)

#### 3.5.2. Typography

Participants also repeatedly discussed typography, which is the style and appearance of the written word, including its use as an image or to be read. Typography discussions were less detailed compared to both color and logo/image discussions. These conversations focused more on the style of lettering. The following excerpts highlight these responses:

“Marlboro and Camel all their lettering is very distinctive for that brand.”

(Group 5, Lower Education)

“Yeah, I guess it doesn’t really stand out. I can’t really describe it, just besides the lettering on it.”

(Group 3, LGB)

Similar to the previous subtheme, when participants discussed typography they also mentioned color. While the use of color, size, and style of lettering was mentioned in participants’ descriptions of packaging, it was not discussed in great detail or focused upon. Typically, typography was described in connection with the color of the package and how the words stood out in contrast to that color.

“It’s blue with white writing on it, and it says, Newport.”

(Group 6, General Population)

“They’re just the plain white with the Kool, the word Kool standing out. It really jumps out at you.”

(Group 4, Mix)

#### 3.5.3. The Pack Itself

Participants identified the physical experience of the cigarette pack as the final meaningful design element. Many participants expressed a strong feeling of loyalty associated with the form of the pack:

“I mean that’s one of the biggest reason that I like these when I get them. It’s because of the rounded pack which they just started doing not too long ago I think and it makes a huge difference.”

(Group 2, Lower Education)

“Whatever I smoke, like when I smoke 100s, its always, always, always in a box. It’s always in a box.”

(Group 1, LGB)

However, the participants also discussed that they were drawn to certain packs as a result of their uniqueness.

“I actually started smoking them for a little while because I thought the little pack was cute. It was really small when it was cute.”

(Group 4, Mix)

### 3.6. Product Brand Experience

Participants shared that cigarette package design elements such as color, logos/images, and type impact their perception and consumption of preferred cigarette brands. Participants also recognized that specific combinations of these elements were used on cigarette packaging, i.e., the design elements worked together as a gestalt. Participants described these branded messages as falling into one of the following design styles: simple and clean, tried and true, or innovative.

#### 3.6.1. Simple and Clean

When participants discussed color and logos, they often spent time describing how some brands had a very basic and straightforward styling. Participants felt that the overall simple style to certain brands’ packaging made it easier for consumers to distinguish and find their brand among the competition.

“I like it. It’s just red and white. It’s simple. I like it. I mean it has like this little gold logo on it. I just like it because it’s just a simple, you just look at it and it looks decent. It’s just simple to look at. There’s not a whole bunch going on the pack.”

(Group 5, Lower Education)

#### 3.6.2. Tried and True

Building on the idea of a simple or clean style is the notion of consistent branding. Participants included in these discussions brands with packaging that have minimal and subtle changes over time.

“They look the same as they do today, exactly the same. They haven’t changed in forty-two years and I believe the reason being is that they’ve been such an iconic mainstream smoker’s choice of brand for a long, long time and they haven’t need to change the packaging because in my eyes, my opinion about Marlboro Reds is that their packaging has always been like a power, like I said before, a power.”

(Group 2, Lower Education)

“Oh, I mean, I don’t think the Newport packaging has changed all that much since they’ve been around. It’s pretty much still the same. You kind of can’t miss them. They’re like Marlboro; you kind of can’t miss the easy identification of it.”

(Group 1, LGB)

Participants respected brands that retained elements of their original packaging style, an approach that participants associated positively with notions of brand longevity, brand power, and a classic cigarette.

#### 3.6.3. Innovative

Conversely, participants recognized and discussed branding styles that were unique and innovative compared to most other brands:

“American Spirit cigarettes, I’ve never tried them. I’ve seen them in the stores. It doesn’t look like a typical pack of cigarettes, as far as the package. I mean, it’s packaged in a typical cigarette box, but the decoration of the box is not typical of a regular cigarette.”

(Group 1, LGB)

“I’ve had people come up to me and go ‘What the heck? What brand is that?’ and I’m like ‘oh, it’s Camel Menthol Silvers’ just because they’ve never seen anything like it before.”

(Group 5, Lower Education)

Most of the discussions about innovative packaging focused on contrasting these styles to boring or typical packages. Participants did not specify what made the packaging innovative. They noted that the overall styling of the package stood out from all the other packs in the marketplace.

## 4. Discussion

### 4.1. Principal Findings

In this study, participants specified several salient design elements that drew attention, provided information about the product, and influenced their cigarette consumption decisions: color, supporting design elements (logos/images, typography, and the pack itself), and a combined product brand experience or gestalt. Participants shared that the combination of these elements on cigarette packaging signaled a particular brand style (simple and clean, tried and true, or innovative), which further influenced their perceptions of various cigarette packs and, in some cases, ultimately influenced purchase behaviors. Changes to cigarette packs can burnish perceptions of a stable “classic” cigarette, or draw consumer attention through the use of novelty in product packaging, fulfilling consumers’ need for uniqueness [30]. This study adds evidence to the argument that changes to the visual design of cigarette packaging can affect consumer behavior and impact the public’s health—and should be regulated [6]. While the global evidence base is strong, we are concerned that industry lawyers may question the external validity of studies conducted outside the U.S. Given decreasing judicial deference to regulators, building a strong evidence base regarding the effect packaging design changes have on consumer responses is critical for the development of regulations about when product design should be considered part of the tobacco product—with any changes reviewed under the full public health standard, as required by the TCA.

Our findings underscore the importance of research to identify the impacts of visual design changes in cigarette packaging. Additionally, our findings demonstrate that both individual design elements and package design, as a whole, influence consumer responses. The constant barrage of industry litigation makes clear that, for the FDA to take any regulatory action to incorporate both individual design elements and package design into a tobacco product’s net assessment under the public health standard, the agency must be armed with sufficient regulatory science. Regulators should consider regulations about meaningful changes to pack design by flagging individual design element changes (e.g., a change in color saturation that is more than X% different) or by creating a system to rapidly test design changes on cognitive and affective mediators (see Crilly [5]) between design and consumer behaviors. Both approaches deserve further consideration.

### 4.2. Study Findings in Context

This study adds rich qualitative data to evidence from tobacco industry documents [6,7,8,9,31], historical research [2,31], assessments of marketing strategies [21], a global study of cigarette packs [32], quantitative U.S. studies linking color and product characteristics [23,33], and extensive evidence from outside of the U.S. demonstrating the impact of plain packaging [24,34,35,36]. Our findings are consistent with this research and with findings from other fields. For example, in the field of consumer behavior, color, contrast, size, and novelty are product design characteristics that influence consumers’ product perceptions—color has been found to be one of the most influential characteristics of product design [18]. The tobacco industry uses a semiotics approach to convey information via cigarette packaging design. Semiotics is the foundation from which designers align marketing messages with the visual elements of their brand to convey a particular meaning to a brand’s consumers. For instance, in our study we found that participants associated particular arrangements of signs (colors, images, logos and/or pack sizes) with specific branded messages (i.e., shared consumer meanings). For example, smokers interpreted the red, white, and hard pack of the Marlboro cigarette as a strong and classic cigarette, as well as a brand for a “white man”. Our findings are, thus, supported by and compatible with the broader marketing, design, and consumer behavior fields.

Our findings also support prior research suggesting that the use of color has effectively evaded FDA’s ban on modified risk descriptors [22,37,38]. Our findings in subtheme 3.3 (color represents taste and strength) show that smokers, themselves, report these connections that prior research has identified in observations of tobacco industry products, reports, and marketing materials [22,37]. Smokers’ self-reports in the International Tobacco Control Policy Evaluation Study show little change in misperceptions about harm from “light” cigarettes after removal of the text descriptors; instead, smokers’ reported greater use of colors and other terms to indicate cigarette strength [38]. Our qualitative data bring smokers’ voices to this body of evidence.

### 4.3. Limitations

Using a qualitative approach prevents generalization to the U.S. population. The telephone-based mode strengthened our approach by including participants from across the U.S., some of whom likely would not travel to an in-person group, and participants without internet access who could not attend a video-based group. However, telephone-based groups have limitations: (a) they preclude the ability to see facial expressions thereby making within-group conversation more difficult; (b) we cannot control the participants’ environment to eliminate other distractions; and (c) related to (b), dropping out is easier (as evidenced in four participants leaving the group before completion). As this study included adult smokers, it does not address the salience of graphic design elements to youth who might be influenced to initiate smoking. Additionally, we focused solely on cigarettes and did not include other tobacco products. Future work should address these important topics.

## 5. Conclusions

Graphic design elements in cigarette packaging convey meaning to adult smokers about the products’ characteristics, which can influence consumer behaviors. Regulation of the visual design of cigarette packaging is warranted under TCA as design may play a significant role in shaping the characteristics of a tobacco product. Future research should examine the impact of packaging design among youth, how to identify when changes to design elements are relevant under a public health standard, and strategies for assessing how visual design changes as a whole, rather than as individual elements, impact consumer behavior.

## Supplementary Materials

The following are available online at www.mdpi.com/1660-4601/14/10/1234/s1, File S1: Focus Group Guide: Semi-Structured.
