# Supplementary material for: “Their Packaging Has Always Been Like a Power”: A Qualitative Study of U.S. Smokers’ Perceptions of Cigarette Pack Visual Design Features to Inform Product Regulation
Source: Int J Environ Res Public Health. 2017 Oct 17;14(10):1234. doi: 10.3390/ijerph14101234 (PMC5664735; doi:10.3390/ijerph14101234)
Supplement: Supplementary file 1 [file ijerph-14-01234-s001.pdf]

Focus Group Guide: Semi-Structured

Hello. Thank you for being part of this conversation today about cigarette packs and their design. I have a few details to share about this project and then we can begin.

You are being asked to participate in a **research** study on cigarette packaging being conducted by Joseph Lee and Paige Averett, faculty members at East Carolina University in the Health Education and Promotion and Social Work departments.

This research will investigate adults' views of different cigarette packs and help us design an experiment. We will record (audio) the focus group discussion. We will not report your name or identity in any way. However, other people in the group may be able to identify you. We are recruiting some groups to have lesbian, gay, bisexual, and transgender participants and some groups to have participants with fewer years of formal education. In those groups, other participants may be able to identify you and know that you were included.

The survey incentives follow your agreement with NORC's AmeriSpeak Panel. We expect this focus group to last an hour to an hour and a half.

Your participation in the research is **voluntary**. You may choose not to answer any or all questions, and you may stop at any time. However, leaving the group may result in not receiving incentives from NORC. Please call or email Joseph Lee at 252-328-4661 or leejose14@ecu.edu for any research related questions or the East Carolina University Office of Research Integrity and Compliance (ORIC) at 252-744-2914 for questions about your rights as a research participant. Does anyone need me to repeat a phone number?

[Introduction and Welcome]

Question 1: [Cognition—Semantic Interpretation Domain] Let's begin by talking generally about various brands and their image, color, size, packaging and <u>what that means about the cigarettes</u>…

- Marlboro
- Newport
- Pall Mall
- American Spirit
    - Utilize follow up prompts as necessary to get group to specifically talk about the packaging and what that says and means—how people then view the cigarettes as a result of the packaging.

Question 2: [Cognition—Symbolic Association Domain] Now lets focus on who smokes these cigarette brands and why?
Go back through brands discussed above and ask the group about the stereotypes and identity of smokers via each cigarette brand.

- Do you think there are there differences in who smokes what cigarettes via race, class, gender, sexual orientation, age?
- Are these differences connected to packaging? How?

Question 3: [Cognition—Symbolic Association Domain] What cigarette brand did you smoke when you first began smoking and what drew you to it?

- Utilize follow up prompts and refocus back to brand and packaging.
- Ask specifically about images, color, size—what did those things say about the brand and about you when you were a new smoker?
- If stories about being given about first cigarettes, re-direct to first purchase.

Question 4: [Cognition—Symbolic Association Domain] What brand do you smoke now and why?

- If the brand changed—why?
  - Utilize follow up prompts back to packaging, size, color.
  - What does this brand say about you now?
- If with same brand—why?

Question 5: [Cognition—Symbolic Association Domain] Have you ever influenced or been influenced by someone in the specific brand to smoke?

- Who and why?
- Utilize follow up prompts and focus back to the specific packaging, identity of who smokes it.

Question 6: [Cognition—Aesthetic Domain] Think back to the last time you purchased cigarettes at a convenience store or just of cigarette packs that you've seen. Tell us about any packs that stood out to you because of the way they looked.

Question 7: [Cognition—Semantic Interpretation Domain] If you didn't care but just wanted a cigarette, what brand would you be willing to smoke? Is there a brand you wouldn't smoke? Why?

- When you join other smokers in a public social outing are you likely to talk and or ask to borrow a cigarette based on the brand they smoke or another factor such as race, gender, etc.
- Are there any specific ways a pack you wouldn't smoke would look?

Question 8: [Affect Domain / Cognition—Semantic Interpretation Domain] Let's think about ________ cigarettes (choose being reflexive of majority brand given by focus group participants)—How would you *feel* if they changed the pack style?

- Have you ever changed cigarettes because they changed the pack style?
- If they wanted to change their look to be more full flavored, what would you change about the pack?
- If they wanted to change their look to show a less-harsh cigarette, what would you change about the pack?
- If they wanted to change their look to show a higher quality cigarette, what would you change about the pack?

Question 9: [Visual Reference Domain] Some designs and marketing can make us think of other things. When you look at ________ cigarettes (choose being reflexive of majority brand given by focus group participants) what does it make you think of? For your own brand?

Question 10: [Affect Domain] Sometimes when we look at an object, its design can make us feel emotions. Has anyone had that experience with a cigarette pack in either a good way (delight, admiration) or a bad way (disgust, boredom)

Question 11: We're nearing the end of this focus group and we've spent a lot of time talking about the design of cigarette packs and what the design means. I'd like to ask you about the Surgeon General's

warning on cigarette packs. What are your thoughts on its design (placement, style, appearance)? What does it make you think of?

Question 12: Is there anything else you would like to share regarding cigarette packaging and how it impacts who smokes a particular brand?
